# Prevalence and Antimicrobial Susceptibility of Multi-Drug Resistant Uropathogens in a Tertiary Hospital in Nepal: A One-Year Audit

**DOI:** 10.31729/jnma.9129

**Published:** 2025-06-30

**Authors:** Manish Man Pradhan, Sangita Sharma, Sujeet Poudyal, Diwas Gnyawali, Suman Adhikari, Suman Chapagain, Bhoj Raj Luitel, Hari Prasad Kattel, Pawan Raj Chalise

**Affiliations:** 1 Department of Urology and Kidney Transplant Surgery, Tribhuvan University Teaching Hospital, Maharajgunj, Kathmandu, Nepal; 2 Department of Microbiology, Tribhuvan University Teaching Hospital, Maharajgunj, Kathmandu, Nepal

**Keywords:** *antimicrobial susceptibility pattern*, *multidrug resistance*, *urine culture*, *urinary tract infection*

## Abstract

**Introduction:**

Antibiotic resistance, especially multidrug resistance, poses a global public health threat. It complicates the treatment of infections like urinary tract infections, leading to treatment failure, extended hospital stays, and increased healthcare costs. Empirical antibiotic therapy, guided by local resistance patterns, is crucial for patient outcomes and infection prevention. This study analyzes urine cultures from TU-Teaching Hospital from January to December 2024 to investigate the prevalence and antimicrobial susceptibility of multidrug-resistant uropathogens.

**Methods:**

This was a retrospective, observational hospital-based study conducted in a tertiary care center after obtaining the ethical approval from Institutional Review Committee (IRC), (Reference number: 470 (6-11) E2). All samples tested fromJanuary 1, 2024, to December 31, 2024 the period included in the study. Data were collected from the Electronic health records of the Microbiology department and analyzed using Microsoft Excel 16.0.0 and SPSS 30.0.0 software to determine the urine culture positivity rate, the prevalence of multidrug-resistant uropathogens, and their antimicrobial susceptibility patterns with a specific focus on resistance to commonly prescribed antibiotics.

**Results:**

A total of 25,315 urine samples were collected for urine culture and sensitivity testing during the study period. Significant bacterial growth was seen in 4,557 (18%). Multidrug resistance was seen in 3,448 (75.66%). The most frequently isolated organisms were Escherichia coli 1724 (50%), Klebsiella 154 (17.7%). *E. coli, Klebsiella, Enterococcus, Citrobacter, Pseudomonas,* and *Acinetobacter* showed resistance to Ceftriaxone (74.61-92.20%), Amoxycillin+Clavulanate (67.58-97%), and Nitrofurantoin (52.29-89.55%) across selectively tested isolates.

**Conclusions:**

Urine samples demonstrated a high prevalence of multidrug resistance to routinely prescribed antibiotics, even among second-line parenterally administered antibiotics. Only costly third-line antibiotics exhibited low resistance.

## INTRODUCTION

The emergence of multidrug-resistant (MDR) bacteria has become a significant public health concern.^1-3^ MDR bacteria are defined as non-susceptible to at least one agent in three or more antimicrobial categories.^4^ Urinary Tract Infection (UTI) are the most prevalent bacterial diseases encountered in tertiary care hospitals, resulting in higher morbidity and mortality rates.^5-8^ Inappropriate prophylaxis and empirical therapy are associated with extended treatment durations, hospital admissions, elevated costs, and increased mortality rates.^2,5,6,9^

Previous studies have reported alarming rates of MDR UTIs, particularly in tertiary hospitals like Tribhuwan University Teaching Hospital (TUTH).^2,7,8,10-16^

Knowing the latest trends in common urinary pathogen growth in urine culture and the pattern of antibiotic sensitivity is crucial for both prophylaxis and the empirical management of UTIs.

Therefore this study was conducted with the objective of investigating the prevalence and antimicrobial susceptibility of multidrug-resistant uropathogens.

## METHODS

This was a retrospective, observational hospital- based study conducted in a hospital in Kathmandu, Nepal, in Department of Urology and the Department of Microbiology at Tribhuwan University Teaching Hospital (TUTH). This study was involved all the urine samples from out from January 1, 2024, to December 31, 2024. The data analysis was completed by March 2025. The target population included all patients (irrespective of age or sex) who submitted urine samples for culture and sensitivity at TUTH during the study period. The sampling frame was all urine samples submitted to the microbiology department, TUTH, within the study period. Sampling unit was each urine sample tested for culture and sensitivity. The census sampling technique was used, with all eligible samples during the defined period being included, and no exclusion criteria were applied.

The total urine samples included was 25,315, and all samples tested during the period were analyzed (no sampling was performed). Data were extracted from: Electronic health records (EHR) of the Microbiology laboratory information system. Data were recorded in a structured collection sheet. Collected variables included: Patient age and gender, urine culture result (positive/negative), organism isolated, antibiotic susceptibility/resistance, and Multi-Drug Resistance (MDR) status. The dependent variable was MDR, which was defined as resistance to one or more agents in three or more antimicrobial classes, and the independent variables were: Age, gender, type of uropathogen isolated: Escherichia coli, Klebsiella, Enterococcus, Pseudomonas, Citrobacter, Acinetobacter, antibiotic resistance status (Sensitive/Resistant) to first-line antibiotics: ciprofloxacin, levofloxacin, cefixime, ceftriaxone; second-line antibiotics: piperacillin- tazobactam, amikacin, meropenem, doxycycline; third-line antibiotics: teicoplanin, tigecycline, colistin, and culture positivity (Yes/No). Data Analysis was done with Microsoft Excel V16.0.0 and SPSS V30.0.0. Descriptive statistics were used, including mean ± SD for normally distributed continuous variables, Median (IQR) for non-normally distributed data, and Frequencies and percentages for categorical variables. The analytical focus was on culture positivity rate, prevalence of multidrug resistance and resistance patterns by organism and antibiotic.

Ethical approval was obtained from the Institutional Review Committee (IRC), Institute of Medicine, Tribhuvan University [Approval reference: 470 (6-confidentiality was strictly maintained through anonymized data. The study adhered to the STROBE (Strengthening the Reporting of Observational Studies in Epidemiology) checklist for observational research.

## RESULTS

During the study period, a total of 25,315 urine samples were collected and processed for culture and sensitivity testing. Of these,13,455 (53.15%) samples were obtained from females and 11,860 (46.85%) were obtained from males, with a median age of 47 (IQR: 28-64). Of these, 14,648 (57.86%) samples were obtained from inpatients, while 10,667 (42.14%) were obtained from outpatients. Of the total samples 4,557 (18.0%) exhibited significant bacterial growth, of which 3,448 (75.66%) had multidrug resistance (MDR). The most frequently isolated pathogens included: Escherichia coli, which was seen in 1724 (50%), Klebsiella species: which were seen in 610 (17.7%), Enterococcus species: in 503 (14.59%), Pseudomonas aeruginosa: in 201 (5.83%), Citrobacter species: in 182 (5.3%), Acinetobacter species: in 79 (2.29%), and Other organism were seen in 149 (4.32%) (Table 1).

**Table 1:** Demographic characteristics of samples undergoing urine culture and drug susceptibility test (n=25315).

Category	N(%)
Total urine samples collected	25,315 (100%)
Females	13,455(53.15%)
Males	11,860(46.85%)
Median age (IQR)	47 years (28-64)
Source of samples	
Inpatients	14,648(57.68%)
Outpatients	10,667(42.14%)
Samples with significant bacterial growth	4,557(18%)
With multidrug resistance (MDR)	3,448(75.66%)
Most frequently isolated pathogens(N = 3448)	
Escherichia coli	1724(50%)
Klebsiella species	610(17.7%)
Enterococcus species	503(14.59%)
Citrobacter species	201(5.83%)
Pseudomonas aeruginosa	182(5.28%)
Acinetobacter species	79(2.29%)
Other organisms	149(4.32%)

**Table 2:** Pathogen-Specific Antibiotic Resistance. (n=25315)

	E. coli	Klebsiella	Enterococcus	Citrobacter	Pseudomonas	Acinetobacter
Amikacin	254(35.47)	154(65.47)	8(72.72)	64(56.14)	152(76.0)	38(55.88)
Amoxycillin+Clavulanate	1112(74.23)	407(76.50)	294(67.58)	155(92.26)	1(100)	67(97)
Ceftriaxone	1355(81.52)	435(74.61)	3(100)	127(72.15)	1(100)	71(92.20)
Chlorammphenicol	15(83.33)	23(92)	1(33.33)	4(80)	1(100	1(88.88)
Colistin Sulphate	1(0.32)	6(2.71)	-	1(1.75)	7(6.6)	-
Cotrimoxazole	1054(61.17)	410(67.76)	4(100)	88(49.16)	2(100)	48(37.79)
Doxycycline	362(55.01)	153(33.33)	43(8.79)	33(29.72)	1(100)	8(12.12)
Meropenem	195(29.23)	294(65.18)	-	49(44.95)	92(69)	36(56.25)
Nitrofurantoin	241(14.82)	431(72.8)	251(52.29)	119(73)	1(100)	60(89.55)
Piperacillin+Tazobactam	753(43.9)	415(67.92)	330(67.20)	113(62.08)	110(55.27)	49(61.25)
Teicoplanin	-	-	11(2.02)	-	-	-
Tigecycline	8 (0.001)	24 (5.49)	1 (0.20)	3 (2.85)	1 (2.85)	4 (7.27)
Vancomycin	-	-	1 (1.86)	-	-	-

Note: Values indicate number of resistant isolates with percentage in parentheses. Antibiotics were tested selectively for each organism; therefore, the denominator (N) varies across antibiotics and organisms. Detailed data are provided in Supplementary File 1.

**Figure 1:**
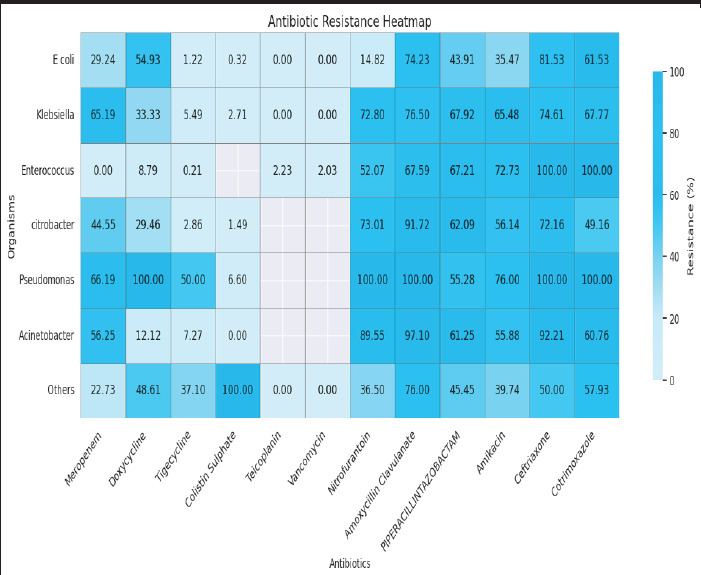
Heat Map of Pathogen-Specific Antibiotic Resistance Patterns (n=25315).

In isolates with significant growth of *Escherichia coli* was seen in the antibiotics like ceftriaxone 1355 (81.52%), amoxicillin-clavulanate 1112 (74.23%), and co-trimoxazole (61.17%). Resistance was seen in doxycycline 362 (55.01%) and piperacillin-tazobactam 753 (43.9%). Resistance was seen in meropenem 195 (29.23%) and nitrofurantoin 241 (14.82%). In isolates with growth of *Klebsiella* resistance was seen in antibiotics like amikacin 154 (65.47%), meropenem 294 (65.18%), ceftriaxone 435 (74.61%), and amoxicillin- clavulanate 407 (76.5%). In isolates with growth of *Enterococcus* species resistance was seen in antibiotics like co-trimoxazole 3 (100%), ceftriaxone 3 (100%), piperacillin-tazobactam 330 (67.2%), and amoxicillin-clavulanate 294 (67.58%) (Table 2) (Figure 1) (Supplementar File 1).

In *Pseudomonas* isolates, amikacin resistance was seen in 152 (76.0%), meropenem in 92 (69%), and piperacillin+tazobactam in 110 (55.27%). Resistance to cotrimoxazole, amoxycillin+clavulanate, ceftriaxone, doxycycline, and nitrofurantoin was recorded in 2 (100%), 1 (100%), 1 (100%), 1 (100%), and 1 (100%), respectively. Resistance to tigecycline was observed in 1 (2.85%), and colistin sulphate in 7 (6.6%). No data were available for teicoplanin and vancomycin (Table 2) (Supplementary File 1).

For *Acinetobacter,* resistance to amoxycillin+clavulanate was reported in 67 (97%), ceftriaxone in 71 (92.20%), and nitrofurantoin in 60 (89.55%). Resistance was observed in 49 (61.25%) for piperacillin+tazobactam, 38 (55.88%) for amikacin, 36 (56.25%) for meropenem, 48 (37.79%) for cotrimoxazole, and 8 (12.12%) for doxycycline. Tigecycline resistance was reported in 4 (7.27%), while data for colistin sulphate, teicoplanin, and vancomycin were not available for this organism (Table 2) (Supplementary file 1).

Among *Citrobacter* isolates, resistance to amoxycillin+clavulanate was reported in 155 (92.26%), ceftriaxone in 127 (72.15%), nitrofurantoin in 119 (73%), and piperacillin+tazobactam in 113 (62.08%). Resistance to amikacin was observed in 64 (56.14%), meropenem in 49 (44.95%), cotrimoxazole in 88 (49.16%), and doxycycline in 33 (29.72%). Tigecycline resistance was noted in 3 (2.85%), and colistin sulphate in 1 (1.75%). Resistance data for Teicoplanin and Vancomycin were not available for *Citrobacter* (Table 2) (Supplementary File 1).

## DISCUSSION

This study revealed an overall culture positivity rate of 18.0% among the submitted samples, which is comparable to a similar study conducted in Nepal, which reported a culture positivity rate of 16.2%. However, it is less than a study by Baral et al. which had found a higher prevalence of culture positivity of 30.8%.^13^ Kumar et al., from India, reported a 25% prevalence of culture positivity among all urine samples tested.^14^ whereas Sharma et al from north-west India had reported even higher 41.8% prevalence.^16^ The reasons for these variations could be attributed to population differences or an increase in screening practices, such as sending urine culture for routine surgical procedures and even for asymptomatic bacteriuria. The majority of isolates were from female patients (53.15%), which aligns with the known higher prevalence of UTIs in reports published elsewhere, which is attributed to anatomical and physiological factors in women.^2^

Escherichia coli was the most predominant pathogen, accounting for 50% of all positive cultures. This is consistent with global and local trends reported by Shakya et al. and Baral et al.^2,13^. Klebsiella species (17.7%) and Enterococcus species (14.6%) were also significant contributors. The presence of Pseudomonas aeruginosa (5.8%) and Acinetobacter species (2.3%) underscores the increasing role of the nosocomial pathogens, particularly in the critically ill or catheterized patients who are admitted to the high care as well as intensive care units of a tertiary care hospital like TU Teaching Hospital.^2,11^

Of all the smaples with significant bacterial growth ; 75.66% of culture-positive isolates exhibited multidrug resistance, which poses a critical challenge in the management of urinary tract infections. This rate is significantly higher than that reported by previous studies conducted in Nepal, which found MDR values of 54% in the study by Shakya et al. and 41% in the study by Baral et al. This high MDR rate may be attributed to the overuse and misuse of broad-spectrum antibiotics, prolonged hospital stays, and inadequate infection control practices.^2,3^

Our findings revealed substantial resistance rates to the commonly prescribed first-line antibiotics like ciprofloxacin, levofloxacin, cefixime, and ceftriaxone, across all major pathogens. This trend questions the efficacy of the empirical therapy that we use and underscores the necessity for updated local antibiograms. Second-line antibiotics also exhibited alarmingly high resistance rates. Resistance to antibiotics like Co-trimoxazole was 61.97%, which is alarming as it has been traditionally utilized for community-acquired urinary tract infections, but now, it may only have a limited clinical utility. Resistance to piperacillin-tazobactam was 55.52%, and that to amikacin was 51.62%, which was also alarming, as these drugs are commonly employed for severe infections, but they have demonstrated diminished effectiveness against these multidrug resistant organisms. Resistance to meropenem (46.11%) is particularly concerning, suggesting the presence of carbapenemase-producing organisms in the community. The level of resistance seen in our study is higher than the reports published previously from Nepal and India.^2,10^ The reason for this could be because of the growing, rampant, and irrational use of antibiotics in the community itself, and as a tertiary care hospital, we get complicated and inadequately treated patients from all over the country.

On the positive side, third-line and reserve antibiotics maintained relatively high sensitivity to the following antibiotics: vancomycin was resistant in only 1.86% of the tested samples, teicoplanin in 2.02%, tigecycline in 3.72%, and colistin sulfate was resistant in 8.48% of the tested samples. These antibiotics remain effective against most multidrug-resistant organisms. This low level of resistance is similar to the report from other studies published.^2,10,14^ However, their reliance is unsustainable due to toxicity profiles, high costs, and the potential for future resistance development; hence, they should be used judiciously.

Escherichia coli maintained high sensitivity to nitrofurantoin of 85.18% in the tested samples, justifying its continued use in uncomplicated lower urinary tract infections. However, it exhibited high resistance to ceftriaxone, which was 81.52%, for amoxicillin clavulanate, it was 74.23%, and for co- trimoxazole, resistance was 61.17%. Moderate resistance was seen for doxycycline, i.e, 55.01%, and for piperacillin-tazobactam, resistance of 43.90% was observed. Klebsiella species exhibited broad resistance, particularly to beta-lactams, aminoglycosides, and carbapenems, which suggests potential extended-spectrum beta-lactamase (ESBL) or carbapenemase production by this organism. Enterococcus species showed preserved sensitivity to vancomycin, with 98.14% of the tested samples being sensitive, and tigecycline sensitivity was very high at 99.80%. However, emerging resistance to nitrofurantoin was seen in 52.29% of the tested samples, and for piperacillin-tazobactam, it was 67.2%. This warrants cautious use, consistent with reports published elsewhere.^8^ Pseudomonas aeruginosa and Acinetobacter species are typically associated with hospital-acquired infections, demonstrating extensive resistance to nearly all antibiotics except teicoplanin and tigecycline, which should be used with utmost caution due to the toxicity associated with these antibiotics.

This high prevalence of multidrug-resistant organisms points towards the need for the immediate and coordinated measures to be implemented in the hospital, such as antibiotic stewardship programs, to reduce empirical use and promote targeted therapy based on culture results. Routine surveillance is essential to update hospital antibiograms and guide empiric prescribing practices. Additionally, infection control interventions should be implemented to reduce transmission, particularly in inpatient settings of the hospital.

## CONCLUSIONS

This study shows that MDR uropathogens, particularly Escherichia coli, Klebsiella, and Enterococcus, cause most of the UTIs. The high prevalence of resistance to first- and second-line antibiotics limits prophylactic and empirical treatment. Third-line antibiotics like tigecycline, colistin sulfate, and teicoplanin remain effective against MDR isolates, but reliance on these last-resort agents is unsustainable and raises concerns about UTI management.
